# Current Perspectives on Cleft Lip and Palate and Children’s Health

**DOI:** 10.3390/children10050857

**Published:** 2023-05-11

**Authors:** Paula Karine Jorge, Eloá Cristina Passucci Ambrosio, Yana Cosendey Toledo Mello-Peixoto, Cleide Felício Carvalho Carrara, Simone Soares, Ana Lucia Pompeia Fraga de Almeida, Maria Aparecida Andrade Moreira Machado, Thais Marchini Oliveira

**Affiliations:** 1 Hospital for Rehabilitation of Craniofacial Anomalies, University of São Paulo, Rua Silvio Marchione 3-20, Bauru 17012-900, Brazil; jorgepk@usp.br (P.K.J.); eloacpambrosio@usp.br (E.C.P.A.);; 2 Department of Pediatric Dentistry, Orthodontics and Public Health, Bauru School of Dentistry, University of São Paulo, Alameda Dr. Octávio Pinheiro Brisolla 9-75, Bauru 17012-901, Brazil; 3 Department of Prosthodontics and Periodontology, Bauru School of Dentistry, University of São Paulo, Alameda Dr. Octávio Pinheiro Brisolla 9-75, Bauru 17012-901, Brazil

Orofacial clefts are the most prevalent craniofacial congenital anomalies, affecting the lip, with or without involvement of the palate, or solely the palate [1,2]. They commonly manifest in isolation; however, they can also be associated with syndromes [3]. These malformations are the result of a multifactorial etiology that prevents the fusion between embryonic facial processes during the fourth and twelfth weeks of intrauterine life [3]. In addition, there are some environmental risk factors related to nutritional deficiencies, hormonal disorders, maternal diabetes, some medications, alcohol intake, and smoking; genetic factors may also be associated with the etiology of cleft lip and palate [4,5,6].

The global estimate of prevalence, according to the World Health Organization, is that one child is affected by cleft lip and palate every 700 births [7]. Regarding the prevalence of phenotypes, there are variations related to gender and ethnicity. Boys are more affected by cleft lip and palate, while girls are more affected by isolated cleft palate [2,3]. These craniofacial anomalies have serious consequences, influencing food, speech, hearing, breathing, craniofacial development, aesthetics, social skills, and, consequently, the well-being and quality of life of individuals and their families [2,5]. For this reason, it is imperative that the rehabilitation protocol begin in the first months of life.

Cleft lip and palate can bring a social stigma that is difficult to approach and define [8]. The gravity of social impairments can be influenced by age and type of cleft [8,9]. Moreover, although cleft lips can cause aesthetic impairments, clefts that involve the palate can also result in speech damage [8].

Moreover, in addition to the social stigma related to speech or aesthetics, there is a social environment in which those children will be raised, which is important for the development of their independence. Families are crucial to supporting the growth and development of children with oral clefts. Because of their anatomical defect, family approaches are either overprotective or permissive in child rearing [10].

In terms of families, there are some aspects that can be employed in this overprotective or permissive rearing, because the new member’s oral cleft affects all family members. There is physical and emotional stress after a cleft diagnosis, which is relevant in their family environment. Furthermore, parents still have sparse information about the rehabilitation process and its impact on their lives [11]. Nevertheless, all this stress will be diminished or even solved after cleft lip and palate repair, leading to a better integration of the cleft patient in society [12].

This Special Issue in *Children*, entitled “Current Perspectives on Cleft Lip and Palate and Children’s Health,” considers all perspectives to children with an orofacial cleft.
